# The Fast Track for Microbiome Research

**DOI:** 10.1016/j.gpb.2019.04.001

**Published:** 2019-04-26

**Authors:** Kang Ning, Yigang Tong

**Affiliations:** 1 MOE Key Laboratory of Molecular Biophysics, Hubei Key Laboratory of Bioinformatics and Molecular-imaging, Department of Bioinformatics and Systems Biology, College of Life Science and Technology, Huazhong University of Science and Technology, Wuhan 430074, China; 2 College of Life Science and Technology, Beijing University of Chemical Technology, Beijing 100029, China; 3 Beijing Advanced Innovation Center for Soft Matter Science and Engineering (BAIC-SM), Beijing University of Chemical Technology. Beijing 100029, China

The microbiome research is undoubtedly one of the most popular topics in biomedical research areas. This is not without reason: given that microbiome is found to be linked with and sometime causes chronic diseases and even cancers, it has become more and more obvious that microbiome plays crucial roles in human health and the surrounding environments.

As the microbiome research moves on a fast track, everyone runs fast. We are delighted to witness novel findings and tools in microbiome research every week, many of which are ground-breaking. However, the track is more like a marathon than a sprint, like any other research area. This means that the progresses could be made step-by-step, while runners also have to be equipped well. That is, we believe applications and research tools in microbiome are both important for the current and future advancement in this field of study.

In this special issue of “Microbiome and Health”, we aim to provide new studies in microbiome that can represent the recent development of applications and tools. Nine articles, all involving microbial communities of humans and in the surrounding environments, are collected, including two review articles and seven original research articles. These articles can be categorized into application-oriented articles and method articles. The application-oriented articles are focused on specific biological questions, utilizing 16S rRNA sequencing or metagenomic data to discover patterns and biomarkers from the microbiome samples. The method articles have used large collections of public data to build computational models that can facilitate in-depth metagenomic analysis.

The application-oriented articles start with a review summarizing the current knowledge on the colonization and development of gut microbiota in early life, with focus on the role of intestinal microbes in pediatric diseases [1]. Li et al. [2] have also put their focus on child health. They have investigated the correlation between gut microbiota of ASD children and their mothers, showing that children with ASD had unique bacterial biomarkers, which would be of importance for the prevention of ASD via microbiota modulation. Shi et al. [3] have investigated the effects of proton pump inhibitors (PPI) on the gastrointestinal microbiota in gastroesophageal reflux disease (GERD), and found that the PPI use in GERD patients is correlated with the changes in gastric mucosal microbiota. In addition, inulin has become one of the most popular supplements for gut microbiota intervention, and Song et al. [4] have investigated the effects of inulin-supplemented diet on gut microbiome as well as on host transcriptome. Their work on mice has shown that the inulin-supplemented diet alleviates glucose and lipid metabolism disorders by modulating the gut microbiota and interacting with host cells in ob/ob mice.

With regard to the impact of humans on microbial communities in surrounding environments, researchers from China and US have jointly investigated microbiota in the Honghu Lake, a freshwater lake in China [5], arguing that agricultural practices can negatively influence not only the physicochemical properties, but also the biodiversity of microbial communities in the lake. In the work by Li et al. [6], the effects of environment factors on microbial communities were also investigated. They showed that short-term antibiotic treatment shifted and diversified the resistome composition, increased the average copy number of antibiotic resistance genes, and increased the potential for horizontal transfer of resistance genes.

Advances of microbiome research also stem from the high-throughput sequencing technologies and big-data mining. Microbial communities can be viewed not only as groups of individual microbes, but also as collections of biochemical functions affecting and responding to an environment or host organism. Metagenomic analysis can identify the genes and pathways carried by a microbial community. Depending on the information required, by using different analytical pipelines and treatments, gene function annotation has already become automatic, self-contained, and effective analytical processes, which can rapidly produce the required output. In this regard, three method articles have been included in this special issue. The first one is also a review article, which has focused on the debate of enterotype, the stratification computational methods, and the application of enterotype on precision medicine [7]. A computational model proposed by Jiang et al. [8] has attempted to find how microbes shape the communities. By proposing a dynamics model of microbial community based on functional genes, they have shown that functional level is important to the assembly of microbial communities. Another method proposed by Li et al. [9] aims to identify bacterial antimicrobial resistance (AMR) genes in metagenomics samples. By using Bayesian framework, they have shown that the method could accurately identify the CNVs and SNVs in AMR genes, with allele fraction significantly changed between the populations.

Nowadays, we have seen tremendous advances in the area of microbiome. Such progress has also been exemplified by these recent studies in this special issue. The field of microbiome has been on its fast track, and the increasing number of research activities conducted would facilitate our understanding of microbiome, health, and environments. These efforts have provided a huge amount of microbiome data, which could be used for better and more in-depth investigations on microbiome in turn, gradually lifting the level of research spirals.

## Competing interests

The authors have declared no competing interests.
